# Performance and discriminatory capacity of Nutri-Score in branded foods in Greece

**DOI:** 10.3389/fnut.2022.993238

**Published:** 2022-09-28

**Authors:** Antonis Vlassopoulos, Alexandra Katidi, Maria Kapsokefalou

**Affiliations:** Laboratory of Chemistry and Food Analysis, Department of Food Science and Human Nutrition, Agricultural University of Athens, Athens, Greece

**Keywords:** Nutri-Score, front-of-pack nutritional labelling, dietary guidelines, food supply, Greece

## Abstract

**Background:**

The harmonization of front-of-pack nutritional declaration is in the heart of food and nutrition policy discussions in Europe. The Nutri-Score system has been proposed by many countries as a potential candidate but its suitability for use across Europe is still under consideration. The current study aimed to evaluate the performance and discriminatory capacity of Nutri-Score in Greece and to test its alignment with the national food-based dietary guidelines.

**Materials and methods:**

Data on the energy, saturated fat, total sugars, sodium, protein, and fiber content per 100°g or ml were extracted for all foods available (*n* = 4,002) in the Greek branded food composition database HelTH. Each food content in fruits, vegetables, pulses, nuts and oils was manually estimated from the ingredients list. The Nutri-Score algorithm was used both as a continuous (FSAm-NPS Score) and a categorical variable [Grades (A)–(E)].

**Results:**

The average FSAm-NPS Score in Greece was 8.52 ± 9.4. More than half of the solid foods (53.8%) were graded from (A) to (C), while most beverages (59.2%) were graded (E). More than 50% of food categories were populated with foods in all Nutri-Score grades, indicative of a good discriminatory capacity. The system scores favorably vegetables, pulses, and low-fat dairy products and unfavourablly sweets and processed meats showing in this way good alignment with the national guidelines. Eggs and seafood scored preferably compared to meat products. Animal fats received less favorable scores and so did cereal products that were highly processed.

**Discussion:**

Nutri-Score showed good capacity to inform consumers toward better food choices in line with the national guidelines. It showed a potential to guide consumers and manufacturers toward less energy dense and more nutrient dense options and highlighted areas of improvement in the food supply.

## Introduction

The creation of a unified Front-of-Pack (FOP) labeling system is at the core of European discussions for the past years (1). Globally, scientists have developed numerous nutrient profiling algorithms over the years and in the past systems like the UK Traffic Lights and the Choices have been implemented in several countries (2).

Front-of-Pack (FOP) labeling has been proposed as a cost-effective tool for consumer education at the point of sale, linked to both improvements in dietary behaviors and in industry practices (3). However, the understanding and use of nutrition labeling varies greatly among European countries and population groups (4). The available evidence points toward color-coded interpretative systems that give an assessment of the healthiness of a specific food as the best option to enable consumer choices (5), but there still a need for guidance and standardization in the design and implementation of such policies (6, 7).

Since 2018, a number of European countries, namely France, Belgium, Germany, Luxembourg, Netherlands, and Spain have decided upon the adoption of Nutri-Score as the new FOP labeling system (8) and the WHO has supported the launch of this system across Europe to promote health (9). The Nutri-Score is a nutrient profiling system developed originally in France, which converts the nutritional content of foods into a five-tier score ranging from A to E (green to red) from healthier to less healthy choices within food groups (10). The system has been tested for its understanding with consumers across 12 countries (11) and there are data available about its capacity to discriminate foods based on their nutritional composition mainly in Central Europe (12). In Southern Europe and the Balkans the data are limited and often in specific food categories (13–15).

The Nutri-Score algorithm, is currently the strongest candidate for application across Europe; however there are concerns raised by some Mediterranean countries that the algorithm has not taken into account the specificities of the food system in the region (16–18). The validation of nutrient profiling systems and especially the assessment of the impact of FOP labeling policies is a data-intensive task. It requires access to granular food composition databases that cover a large number of foods currently sold in the country or region of interest and a representation of multiple food groups rather than just one food category or subcategory. Latest reports are focused on central and Western Europe exploiting regional branded food composition databases (BFCDs) available in the EuroFIR platform and in Open Food Facts (11). In Southern Europe and the Balkans, access to that type of data is still limited and hence there is a gap in the evaluation of the Nutri-Score in the region.

A new tool that could facilitate the investigation of Nurti-Score in Greece, is the Hellenic Food Thesaurus (HelTH). HelTH is a BFCD launched in 2019 by the Agricultural University of Athens, which collects and analyses all nutritional and quality data provided on labels of branded food products (19).

The aim of the present study was to assess the performance and test the discriminatory capacity of the Nutri-Score algorithm in the Greek foodscape, as well as to evaluate the alignment of Nutri-Score with the national food-based dietary guidelines (FBDGs) of Greece.

## Materials and methods

### The Hellenic food thesaurus database

Food composition data were extracted from HelTH, a dynamic dataset that compiles data on the nutritional composition and quality characteristics of branded foods available in Greek supermarkets.

Hellenic food thesaurus (HelTH) started as an initiative in 2018 and in its first version (11/2019), used in this analysis, contained data for *n* = 4,002 food products. In brief, HelTH includes information on the nutritional composition of foods, extracted from food labels available on the e-shops of large supermarket chains in Greece. Data on any health and/or nutrition claims made on pack, and information on any other quality claims written on pack (environmental claims, logos, origin, etc.) and the nutritional declaration was checked for quality by two independent researchers and curated in the database. A detailed description of the methodology and structure of HelTH has been published previously (19).

Data were selected on the basis of the availability of nutritional composition data and the availability of data to calculate Nutri-Score. Herbs and spices, alcoholic beverages, dietary supplements, and foods for special nutritional use were excluded (*n* = 139) as they are not included in the scope of the Nutri-Score according to the European regulation (10). All information around the nutritional composition was taken from the packaging and entered into the database. All products were classified in 13 categories and 36 subcategories following the LanguaL methodology.

All data of the HelTH BFCD were checked and cleaned. In particular, duplicates of the same product, constituting part of an offer or discount multi-package, or by human error appearing twice at the online platform, were excluded (multi-pack items were deleted where the single item was also available).

### Nutri-Score calculation

The latest Nutri-Score algorithm was used in this analysis (20). In brief, the FSAm-NPS score was calculated for each food based on their nutritional composition per 100°g/ml of food/beverage (20). For each food, content of energy (kJ), total sugars (g), saturated fatty acids (SFAs) (g), and sodium (mg) were considered “negative nutrients” scored from 0 to 10 with higher scores for higher content. In the case of added fats, instead of SFA content the Ratio of SFA/Total Fat was used. Protein content (g), fiber content (g), and fruits/vegetables/pulses/nuts/specific oils content (FV%) were considered “positive nutrients” and received points from 0 to 5 with higher scores for higher content.

An overall score ranging from −15 to +40 was calculated by subtracting the “positive nutrients” score from the “negative nutrients” score. More specifically, fiber and FV scores were subtracted for all products, but the protein score was subtracted only in products with “negative nutrients” score < 11, those with an FV score > 5 or for cheeses.

The FSAm-NPS score was translated to Nutri-Score based on the following criteria (20): (A) was assigned to solid foods with a score from −15 to −1 or waters, (B) to solid foods with a score from 0 to 2 and beverages from −15 to 1, (C) to solid foods with a score 3 to 10 and beverages from 2 to 5, (D) to solid foods from 11 to 18 and beverages from 6 to 9 and (E) to solid foods from 19 to 40 and beverages from 10 to 40.

All nutrient contents were based on the labeled nutritional composition declaration. FV% was estimated based on the ingredient list in a two-step process. Firstly, all foods were screened to assess the presence of at least 40% content in fruits, vegetables, pulses, nuts and rapeseed, walnut, and olive oils, which is the minimum content required. Then for the products that met this minimum requirement a thorough quantification was carried out.

For the purpose of the study, products that did not contain any data about their energy, saturated fat, total sugar or sodium content (*n* = 778) were excluded, as no Nutri-Score could be calculated. Missing nutrient values could be due to lack of nutritional declaration or low-quality images obtained from the specific foods. On the contrary for “positive nutrients” missing information were imputed as zero.

### Evaluation of alignment with the national food-based dietary guidelines

The latest FBDGs were developed in 2014 from a group of experts, have been endorsed by the National Nutrition Policy Committee and adopted by the Greek Ministry of Health on October 2017 as the national food-based dietary guidelines. These guidelines cover all age groups, but for this analysis only the parts on non-pregnant, healthy adults were used as a reference (21). To test the alignment between Nutri-Score and the national FBDGs (21), Langual food categories and subcategories were matched to the food categories as mentioned in the guidelines. The national FBDGs provide food-based guidance on the basis of “foods to avoid,” “foods to consume in moderation” and “foods to promote.” For the purpose of this analysis it was assumed that Nutri-Score grades (A) or (B) represented “foods to promote,” grade (C) represented “foods to consume in moderation,” and grades (D) and (E) represented “foods to avoid,” following previously published methodology (22).

The national FBDGs provide an overarching guidance to avoid energy-dense foods and prefer nutrient-dense options. As the Nutri-Score algorithm follows a similar methodology in its ranking algorithm it was considered that for the algorithm to be considered aligned with the guidelines, within each food category there should be evidence of foods being ranked in multiple grades rather than all foods being clustered in a single grade.

In the same context, the national FBDGs advice toward food choices that is poorer in total fat, SFA, added sugars, salt and richer in unrefined cereals and fiber. To test the alignment of Nutri-Score with this guidance the macronutrient distribution of energy, SFA, salt and total sugars across Nutri-Score grades within each food category were tested. In the case of promoting the consumption of unrefined cereals, although the guidelines call for the promotion of wholegrain cereals, Nutri-Score does not track wholegrains and HelTH does not include wholegrain content data. As such in this analysis a food’s fiber content was used as a proxy for wholegrain content. In this analysis, a decreasing fiber content with each increasing Nutri-Score grade would be considered an alignment to the FBDGs.

### Statistical analysis

Statistical analysis was carried out using IBM SPSS Statistics ^®^ (version 23, Northridge, CA, USA). Nutritional composition data (content per 100 g or 100 mL of product) and the FSAm-NPS score were analyzed as continuous variables. Data were tested for normality using the Kolmogorov-Smirnov test. None of the variables followed the normal distribution. Therefore, variables were expressed as median (interquartile range). We assessed the distribution of prepacked products across different NS grades for main categories and subcategories and displayed this information in boxplots emphasizing median, 25th, and 75th percentiles. Discriminating ability was considered good when the food group comprised at least three different NS grades (12, 13). Differences were tested using the Kruskal-Wallis non-parametric test for k independent samples. Between-group differences were tested using the Mann-Whitney *U* test for continuous variables. Statistical significance was set at 0.01% to adjust for multiple comparisons (Bonferroni correction).

## Results

### Distribution of Nutri-Score

A total of 3,224 products were included in the final analysis with grain and grain products being the largest food category followed by dairy products and imitations and then non-milk beverages, sugar products and miscellaneous foods (Table 1). The median FSAm- NPS score for all categories was 10, with significant differences among the various food categories (*p* < 0.001). Vegetables had the lowest average score among all groups (*p* < 0.001, data not shown), followed by ready meals, eggs, and fruits which all received similar Nutri-Score (*p* = 0.39, data not shown). Sugar products had the highest FSAm-NPS Score compared to all food categories (*p* < 0.001 data not shown), followed by meat products, fats and oils, miscellaneous foods and non-milk beverages (*p* < 0.001 with the remaining categories, *p* > 0.05 among them, data not shown).

**TABLE 1:** Mean Nutri-Score per food category in the *n* = 3,224 branded food products of the Hellenic food thesaurus (HelTH) branded food composition databases (BFCD) analyzed.

Food category	Nutri-Score median (Q1, Q3)
Milk, milk products, and substitutes (*n* = 574)	3 (0, 15)
Eggs or egg products (*n* = 30)	0 (−1, 0)
Meat or meat products (*n* = 103)	15,5 (11, 19)
Fish and seafood (*n* = 58)	5 (2, 14)
Fats and oils (*n* = 63)	13 (9, 19)
Grains or grain products (*n* = 935)	9 (−1, 15)
Nuts and seeds (*n* = 114)	8 (2,13)
Vegetables or vegetable products (*n* = 210)	−6 (−10, −5)
Fruits or fruit products (*n* = 37)	1 (−2, 4)
Sugar or sugar products (*n* = 288)	22 (14, 26)
Non-milk beverages (*n* = 370)	11 (5, 15)
Ready meals (*n* = 76)	−0.5 (−4, 2)
Miscellaneous (*n* = 278)	11 (5,15)
Total (*n* = 3,085)	10 (0, 16)

**FIGURE 1:**
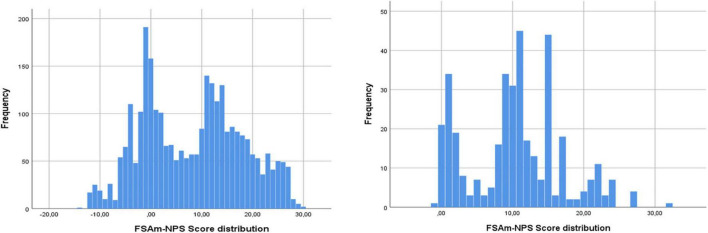
Distribution of FSAm-NPS Score among branded solid foods and beverages in the He1TH FCDB (*n* = 3,224).

The distribution of FSAm-NPS Score across all categories is shown in Figure 1, separately for solids and beverages. Overall, 21.0% all of foods were rated A, 13.0% B, 16.5% C, 27.9% D, and 21.6% E. The distribution shows spikes especially around in-between Nutri-Score grades. For example, 6.7 and 5.5% of solid products were graded with score −1 (Grade A) and with score 0 (Grade B). The next highest prevalence 4.9% was seen around score 11 (start of Grade D).

**FIGURE 2:**
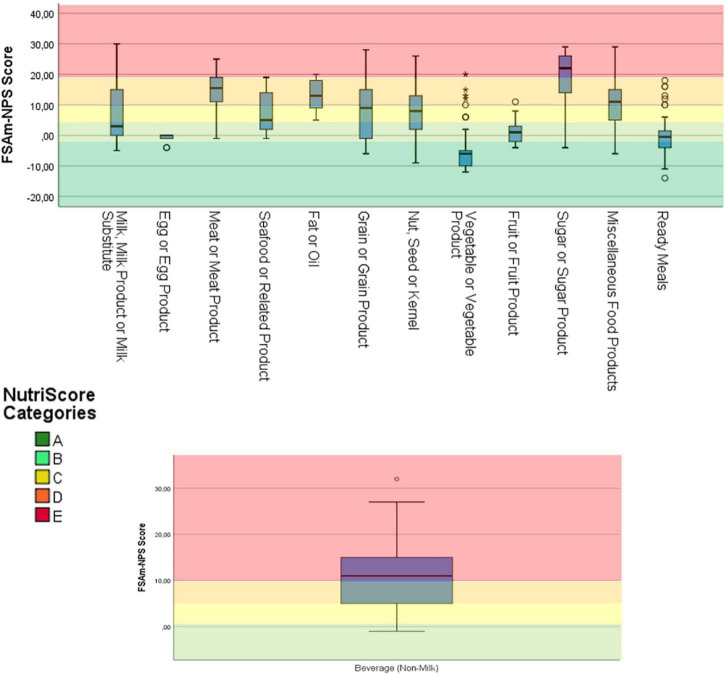
Overall distribution of products within the main food categories. Dark green: Nutri-Score “A”, light green: Nutri-Score “B”, yellow: Nutri-Score “C”, light orange: Nutri-Score “D”, and dark orange: Nutri-Score “E”. No Nutri-Score “A” was represented on the graphic of beverages, given that only waters can be classified as “A” and were thus excluded from the graphic (*n* = 3,224).

Overall, 100% of egg products, 95.2% of vegetables products, 77.6% of ready meals, 67.5% of fruit products, and 48.6% of milk and milk products categories were graded as (A) or (B). On the contrary, 85.8% of meat products, 90.3% of sugar products, 65.1% of fats and oils, and 56.8% of miscellaneous foods were graded as (D) or (E). The same was true for beverages with 74.9% of all beverages being graded as (E) (Figure 2, Table 2).

**TABLE 2:** Nutri-Score distribution in food subcategories (*n* = 3,224) of the Hellenic food thesaurus (HelTH) branded food composition databases (BFCD).

Food category	Food subcategory	A n (%)	B n (%)	C n (%)	D n (%)	E n (%)
Milk, milk products, and substitutes (*n* = 574)	Milk (*n* = 147)	51 (34.7)	86 (58.8)	6 (4.1)	2 (1.4)	2 (1.4)
	Yogurt (*n* = 152)	78 (51.3)	54 (35.5)	20 (13.2)	–	–
	Cheese (*n* = 159)	2 (1.3)	3 (1.9)	15 (9.4)	125 (78.6)	14 (8.8)
	Cream (*n* = 30)	–	2 (6.7)	7 (23.3)	21 (70.0)	–
	Frozen dairy desserts (*n* = 38)	1 (2.6)	1 (2.6)	2 (5.3)	23 (60.5)	11 (28.9)
	Imitation milk products (*n* = 48)	–	1 (2.1)	23 (47.9)	15 (31.3)	9 (18.8)
Eggs or egg products (*n* = 30)		11 (36.7)	19 (63.3)	–	–	–
Meat or meat products (*n* = 105)	Preserved meat (*n* = 68)	–	1 (1.5)	10 (14.7)	37 (41.6)	20 (29.4)
	Sausages (*n* = 21)	–	–	–	12 (57.1)	9 (42.9)
	Meat dish (*n* = 16)	1 (6.3)	2 (12.5)	1 (6.3)	12 (61.5)	–
Fish and seafood (*n* = 58)		4 (6.9)	15 (25.9)	14 (24.1)	22 (37.9)	3 (5.2)
Fats and oils (*n* = 63)	Margarine or mixed fats/oils (*n* = 38)	–	–	21 (55.3)	17 (44.7)	–
	Animal fat/butter (*n* = 25)	–	–	1 (4.0)	8 (32.0)	16 (64.0)
Grains or grain products (*n* = 935)	Cereal or cereal-like milling products (*n* = 36)	4 (11.1)	5 (13.9)	7 (19.4)	13 (36.1)	7 (19.4)
	Rice (*n* = 63)	26 (41.3)	18 (28.6)	10 (10.3)	5 (7.9)	4 (6.3)
	Pasta (*n* = 201)	172 (85.6)	6 (3.0)	10 (5.0)	13 (6.5)	–
	Breakfast cereal and bars (*n* = 152)	18 (11.6)	13 (8.6)	62 (40.8)	59 (38.8)	–
	Bread or similar products (*n* = 180)	33 (18.3)	45 (25.0)	54 (30.0)	35 (19.4)	13 (7.2)
	Fine bakery ware (*n* = 227)	–	1 (0.4)	18 (7.9)	87 (38.3)	121 (53.3)
	Savory cereal dish (n = 76)	–	1 (1.3)	19 (25.0)	51 (67.1)	5 (6.6)
Nuts and seeds (*n* = 114)	Nuts (*n* = 54)	17 (31.5)	14 (25.9)	18 (33.3)	3 (5.6)	2 (3.7)
	Seeds (*n* = 34)	–	1 (2.9)	10 (29.4)	22 (64.7)	1 (2.9)
	Nuts or seeds products (*n* = 26)	–	–	5 (18.5)	19 (73.1)	2 (7.7)
Vegetables or vegetable products (*n* = 210)	Vegetables (*n* = 149)	135 (90.6)	4 (2.7)	5 (3.4)	4 (2.7)	1 (0.7)
	Starchy roots (*n* = 17)	10 (58.8)	7 (41.2)	–	–	–
	Pulses and products (*n* = 44)	43 (97.7)	1 (2.3)	–	–	–
Fruits or fruit products (*n* = 37)		15 (40.5)	10 (27.0)	11 (29.7)	1 (2.7)	–
Sugar or sugar products (*n* = 288)	Jams (*n* = 56)	2 (3.6)	–	21 (37.5)	32 (57.1)	1 (1.8)
	Non-chocolate confectionary (*n* = 40)	1 (2.5)	2 (5.0)	1 (2.5)	34 (85.0)	2 (5.0)
	Chocolate (*n* = 192)	–	–	1 (0.6)	19 (9.9)	172 (89.6)
Ready meals (*n* = 76)	Ready-to-eat (*n* = 43)	11 (30.6)	14 (38.9)	8 (22.2)	3 (8.3)	–
	Frozen, semi-ready (*n* = 41)	27 (67.5)	7 (17.5)	4 (10.0)	2 (5.0)	–
Miscellaneous (*n* = 278)	Spice, condiment (*n* = 144)	10 (4.4)	20 (8.7)	78 (34.1)	74 (32.3)	47 (20.5)
	Prepared food product (*n* = 135)	5 (3.7)	10 (7.5)	34 (25.4)	70 (52.2)	15 (11.2)
Non-milk beverages (*n* = 370)	Juice/nectar (*n* = 157)	–	1 (0.6)	10 (6.4)	48 (30.6)	98 (62.4)
	Non-alcoholic beverages (*n* = 213)	–	55 (25.8)	27 (12.7)	10 (4.7)	121 (56.8)

In 8 out of the 13 food categories there was at least one product in every Nutri-Score grade. The categories with the lowest variability were egg products and fats and oils, in that order.

In terms of the distribution in subcategories within the milk products, milk and yogurt had the highest number of products rated (A) or (B), 93.5 and 86.8% respectively. On the other hand, cheeses were graded mostly (D) (78.6%), however a small proportion of cheeses (< 2%) were graded (A) and (B) (Table 2). Imitation milk products received an overall positive Nutri-Score with 79.2% being graded (A) or (B).

For meat products, the most common Nutri-Score was (D) for all subcategories. Preserved meats and meat dishes showed some variability in Nutri-Score but the absolute numbers are very small (*n* < 10) (Table 2). In the case of fats and oils, animal sources were at large (96%) graded as (D) or (E), while plant-based margarines were graded either (C) or (D). At this point is worth mentioning that no vegetable fats were available in the version of HelTH used for the current analysis.

Grains and grain products, the largest food category, showed great variability in Nutri-Score. As the largest and most versatile food category, this variability was also seen among its subcategories with pasta, breads, rice, and cereal milling products receiving Nutri-Scores across the spectrum with larger numbers at the lower scores (Table 2). On the other hand, fine bakery ware and savory cereal dishes had a Nutri-Score distribution that technically started from grade (C) onward. For vegetables, the distributions were all skewed toward grades (A) and (B) for all vegetables, starchy or not, and for pulses alike. Some vegetable products existed with Nutri-Score grades above (C) but those represented less than 5% of the subcategory (Table 2). In contrary to vegetables, processed fruit products graded as (C) or (D) were 32.4% of all foods in the category.

Jams, non-chocolate confectionary, condiments and prepared food products were subcategories where Nutri-Score (D) was the dominant grade and that was more pronounced in the sweet options rather than the savory options. In general, those subcategories represent mainly sweet and savory snack foods. Sweet snacks are predominantly graded as (D) with the exception of chocolates with 89.6% of all products graded as (E). For savory snacks the main volume of products is split between grades (C) and (D).

More complex food products as they are represented by the composite dishes in the ready-to-eat and frozen foods subcategories receive overall positive grades, with > 70% of all products in (A) and (B). Semi-prepared dishes performed better than ready-to-eat products but even then, ready-to-eat foods were equally split between (A) and (B).

Finally, almost all juices and nectars (98%) were graded as (E), while for the remaining non-alcoholic beverages a quarter of the products were graded as (B) which is the lowest Nutri-Score for beverages other than water.

### Alignment with food-based dietary guidelines

In terms of agreement with the national food-based guidelines, Nutri-Score shows a preliminary good agreement as shown in Table 3. Overall, food groups like vegetables, fruits, and pulses that are mentioned in a positive manner in the guidelines are also scored preferably by Nutri-Score. On the other hand, animal sources of protein are more strictly judged by the system. From animal protein sources, Nutri-Score shows a tendency to favor eggs and seafood and to unfavour processed and cured meat products, in line with the national FBDGs. Increasing Nutri-Score in meat products and seafood was associated with higher sodium and SFA content (*p* < 0.001, data not shown). Sweets are overall unflavored and graded as (D) and (E), as are juices.

**TABLE 3:** Presentation of Greek food-based dietary guidelines for adults (21) per food group/subgroup and the relevant distribution of Nutri-Score calculated for the branded food products of the Hellenic food thesaurus (HelTH) branded food composition databases (BFCD) (*n* = 3,224).

Food group/Subgroup	Guideline	A n (%)	B n (%)	C n (%)	D n (%)	E n (%)
Vegetables	Consume 4 portions a day Prefer fresh and uncooked vegetables Consume vegetable based main dishes 1–2 times/week	135 (90.6)	4 (2.7)	5 (3.4)	4 (2.7)	1 (0.7)
Fruits	Prefer fresh fruits Consume dried fruits in moderation Avoid canned fruit especially if preserved in syrup	15 (40.5)	10 (27.0)	11 (29.7)	1 (2.7)	–
Juices	Prefer fresh fruits to juices and consume up to 1/2 cup a day Avoid prepacked juices	–	1 (0.6)	10 (6.4)	48 (30.6)	98 (62.4)
Cereals^1^	Prefer wholegrain cereals, pasta and rice When choosing bread and breakfast cereals read the labels carefully as they can be hidden sources of salt and/or sugars	253 (39.7)	88 (13.8)	92 (14.4)	176 (27.6)	29 (4.5)
Potatoes	Consume 3 times a week Avoid French fries	10 (58.8)	7 (41.2)	–	–	–
Dairy products	Consume 2 portion/day with preference toward low fat milk, low fat yogurt and low-fat cheese Prefer foods naturally lower in fat and sodium	146 (25.4)	170 (29.6)	54 (9.4)	171 (29.8)	33 (5.7)
Milk	Prefer low fat milk Avoid sugar sweetened milk	51 (34.7)	86 (58.8)	6 (4.1)	2 (1.4)	2 (1.4)
Cheese	Prefer low fat and low sodium cheese	2 (1.3)	3 (1.9)	15 (9.4)	125 (78.6)	14 (8.8)
Yogurt	Prefer low fat yogurt	78 (51.3)	54 (35.5)	20 (13.2)	–	–
Cream	Avoid cream and replace it with yogurt when possible	–	2 (6.7)	7 (23.3)	21 (70.0)	–
Pulses	Consume 3 times/week Source of plant protein, fiber and micronutrients	40 (90.9)	2 (4.5)	2 (4.5)	–	–
Eggs	Consume up to 4 eggs/week Source of high-quality protein	11 (36.7)	19 (63.3)	–	–	–
Meat	Consume up to 1 portion/week red meat Consume 1–2 portions/week white meat Avoid processed or cured meats	1 (0.9)	3 (2.8)	11 (10.4)	61 (57.5)	30 (28.3)
Fish and seafood	Consume 2–3 portions/week Prefer fresh fish to seafood Avoid any processed fish/seafood	4 (6.9)	15 (25.9)	14 (24.1)	22 (37.9)	3 (5.2)
Fats and oils	Consume all fats and oils in moderation (total 4–5 portions/day) Prefer olive oil as the main oil followed by other vegetable oils (except palm oil) Avoid animal fats and hard margarines	–	–	22 (34.9)	25 (39.7)	16 (25.4)
Nuts and products	Consume in moderation Count toward the 4–5 portions/day of fats and oils Prefer unsalted nuts Use nut spreads as a snack	–	–	15 (25.0)	41 (68.3)	4 (6.7)
Sweets^2^	Reduce all sweets to 1 portion/week	3 (0.6)	3 (0.6)	34 (6.6)	179 (34.8)	296 (57.5)
Spices and condiments	Avoid commercial spices and condiments as they are sources of sodium and sugar	1 (0.7)	6 (4.2)	59 (41.0)	63 (43.8)	15 (10.4)
Beverages	Prefer water and unsweetened beverages Avoid sugar-sweetened beverages	–	55 (25.8)	27 (12.7)	10 (4.7)	121 (56.8)

^1^Not including fine bakery ware.

^2^Including fine bakery ware.

Fats, oils and nuts are mentioned as food groups to be consumed in moderation and with close consideration in their nutritional composition, in the case of Nutri-Score grading all food groups that contained the statement in moderation did not receive any grade below (C), which could be considered in agreement with the guideline. In the case of grains and cereals, Nutri-Score showed a wide variability but an analysis of the fiber content showed that foods graded as (D) and (E) had significantly lower fiber content compared to all other Nutri-Score grades (*p* < 0.01). More specifically cereal products in Nutri-Score grades (D) and (E) had an average fiber content of 3.91 ± 2.7 and 2.50 ± 1.2 g/100°g respectively, as opposed to products graded (A) to (C) which had an average content of 5.24 ± 4.6 g/100°g. The majority of wholegrain/non-refined cereals (76%) were graded either (A) or (B) which indicates a greater capacity to highlight the differences in the nutritional composition of this subcategory.

The Greek food-based guidelines mention a preference toward dairy foods that are low in fat naturally, meaning a prioritization of milks, yogurts which is documented in the Nutri-Score performance in the dairy subcategories. Only a small number of cheeses were graded as (A) or (B) but there was good discriminatory capacity among cheeses as all Nutri-Score grades were populated. In dairy products, increasing Nutri-Score was associated with increasing energy, SFA, sodium, and total sugars content (*p* < 0.001, data not shown). On the other hand, although the National FBGs include a mention on avoiding sweetened dairy products, only a few products (namely sweetened condensed milk) received a Nutri-Score grade above (C). Sweetened yogurts (either kid’s yogurts or yogurt desserts) were graded as (B) or (C) even when sweetened with fruit juices/jams.

## Discussion

This study is the first to apply the Nutri-Score algorithm in a large sample of branded food products currently available in Greece. In that context this study also expands previous work on the application of Nutri-Score in countries of the European south and to test its alignment with the national food-based dietary guidelines (23).

### Distribution and discriminatory capacity of Nutri-Score

The overall aim of Nutri-Score is to facilitate consumers’ understanding of the nutritional information and thus to help them in making informed choices (20). For this to be achieved Nutri-Score needs to be able to identify alternatives within the same food group. In the current analysis ∼50% of all food groups were populated with products that were graded across the whole Nutri-Score spectrum (A)–(E). In fact, only three food groups, eggs, juices, and fats and oils showed narrow distributions. The discriminatory capacity of Nutri-Score was less apparent in subcategories, ∼44% of the subcategories covered all the Nutri-Score range. Larger categories and subcategories showed better discriminatory capacity and on the opposite side very homogeneous categories showed limited discriminatory capacity. These results are in agreement with previous reports from various European countries (12, 13, 22) but also from Mediterranean countries like Italy and Spain (23).

When the FSAm-NPS score variability is studied it becomes apparent that there is a clustering of products around cut-off values, indicating that Nutri-Score once rolled out could be used as a stimulus for food reformulation. In fact, the highest clustering of food products is seen in the cut-off value between grades (B) and (C) (FSAm-NPS Score = 1) with a second peak at FSAm-NPS Score = 11, the cut-off point between grades (C) and (D). That shows that although currently 27.9% of all products are graded (D) and 16.5% are graded (C), it is possible for a substantial proportion of those foods to improve without extensive reformulation. In fact, 9.5% of all foods have an FSAm-NPS score = 11–12 and 7.1% of all foods have scores at FSAm-NPS = 1–2. Similar results were seen in the Netherlands (22) in France (24) were the potential of Nutri-Score to guide reformulation was deemed high. The phenomenon of clustering around cut-off points is documented in multiple countries across Europe (13, 23) but most importantly it is more apparent in countries with higher average Nutri-Score. Overall, the FSAm-NPS Score in Europe ranges for 7.6–9.9, with Slovenia, France reporting the highest scores (13, 23). However, there is a positive association between the number of foods analyzed and the average Nutri-Score for the country (23). This could be explained by the type of data included in each analysis, the same analysis when performed in branded food composition databases only leads to greater average FSAm-NPS scores as compared to analyses carried out using a combination of branded and generic food composition databases (12, 23). In that context as Nutri-Score is designed to be implemented on packed foods, one could argue that branded food composition databases are more appropriate to test the algorithm’s performance in conditions that mimic the foodscape. In fact, an analysis in the Slovenian foodscape highlighted that branded food composition data combining with market share data are even more appropriate to describe the performance of Nutri-Score as often the products with the less desirable nutritional compositions are the ones that are preferred from the consumers (13).

In the case of Greece, Nutri-Score managed to successfully identify “healthier” options for consumers in all food categories and subcategories allowing for product substitutions up to two Nutri-Score grades below. The agreement of our findings with previous analyses in other countries also adds to the discussion of the potential for extrapolation of the findings across Europe and even in other regions, suggesting that Nutri-Score performance is rather homogenous in multiple settings.

### Alignment with the national food-based dietary guidelines

As FOP labeling’s main purpose is consumer information on healthier food choices, a key stage in its validation is testing its alignment with national and international guidelines (7, 25, 26). In the past, Nutri-Score has been validated against the dietary guidelines of various countries (10, 12, 13, 23), while some controversies were raised in others, like in the Netherlands (22, 27). In our analysis of the alignment of Nutri-Score with the food-based dietary guidelines for Greece we found good agreement between the two both in principle (nutrients to be reduced, nutrients to be promoted) but also among specific subgroups. Overall, all food groups that were mentioned in the guidelines as foods to be promoted like vegetables, fruits and pulses received the lowest Nutri-Score. Although the guidelines mention fruits as foods to be promoted in our analysis approximately 30% of all foods were graded (C). This can be explained from the nature of the foods available in HelTH, which in the case of fruits would include mainly dried and canned fruit (19). In this context, the Nutri-Score outcome in this analysis reflects quite closely the spirit of guidelines, that call for an increased intake of fresh fruit, the consumption of dried fruit in moderation and avoidance of canned fruit and fruit juices (21).

A similar explanation could be offered for the unfavorable grading of the meat and meat products group, which in the case of HelTH is mainly populated by sausages, cured or dried meats which are discouraged both as potential carcinogens and for their high fat and sodium content (21, 28). When studied collectively in our analysis, animal protein sources like eggs and seafood were prioritized by the Nutri-Score algorithm over processed meat. Plant based protein from pulses were even further promoted. In the case of ready meals, that was also true as meals higher in protein but poorer SFA and sodium received better FSAm-NPS score, directing consumers toward white meat and fish/seafood options. Although not covered by the national guidelines, even among dairy products, plant based dairy imitations also received better FSAm-NPS Scores. For dairy products, the Nutri-Score algorithm showed good alignment with the guidelines asking for a prioritization over lower fat and sodium dairy options such as milk and yogurt and then the consumption of cheeses that are naturally low in fat. In our analysis we were able to identify a small number of such products, both traditional and low-fat versions of traditional foods. Previous work target in the most commonly consumed traditional Greek cheeses, confirmed epidemiological data suggesting that traditional cheeses are generally discouraged by Nutri-Score (14, 15) but there might be a need for a targeted expansion of such databases to include less popular traditional cheeses that are naturally low in fat and/or sodium (29). In the case of sweetened dairy, Nutri-Score graded sweetened yogurts as (B) or (C), as opposed to (A) for the low fat, unsweetened alternatives. As far as within category comparisons are concerned the algorithm shows a fair discriminatory capacity between the sweetened and unsweetened variant. The discriminatory capacity is stronger across categories when comparisons between sweetened dairy products and sweets and confectionaries are concerned. The Nutri-Score algorithm also shows a good capacity to differentiate refined and non-refined cereal as non-refined cereals were in their majority graded as (A) or (B) and were all concentrated in the lower part of the FSAm-NPS distribution.

The lowest discriminatory capacity was seen among sweets and more so among chocolates. Although discriminatory capacity is always better to help identify “healthier” options in the case of those food subcategories, the lack of discriminatory capacity is in line with national and international guidelines that call for a reduction in sugar intake and the avoidance of sweets to a maximum of one portion per week (21, 30).

Finally, a key consideration for Nutri-Score in Greece is its performance vis-à-vis fats and oils. In the case of our analysis, the dataset used did not include any data on vegetable oils (19), as such the results presented herein do not include any data on vegetable oils including olive oil. On the contrary the dataset includes data on vegetable and animal fats. As per the latest Nutri-Score algorithm (20), olive oil is automatically graded as (C). With this in mind, there are two important considerations in the topic, with the first being the discriminatory capacity of the algorithm. Based on our data, no fats available in our dataset received a Nutri-Score grade lower than (C), margarines received either (C) or (D), while animal fats were primarily graded as (E). That indicates an agreement with the FBDGs that propose the avoidance of animal fats and the larger uptake of vegetable fats and oils. As far as, olive oil is concerned it is true that it is not graded more favorably than all fats available in the Greek marketplace and in fact a large proportion of margarines would receive a similar Nutri-Score to olive oil. It is important that future research performs more targeted analysis in fats and oils, in order to understand whether there is still a need for further finetuning of the algorithm although preliminary data suggest against it (17). The second issue is linked with the nutritional composition of the fats that are graded similarly to olive oil. A preliminary analysis of our data, indicates that the majority of margarines with a Nutri-Score (C), are reformulated products with higher olive oil content, or fortified with plant-sterols, or even products with higher protein content (yogurt fortified margarines). Overall, the caping of Nutri-Score at grade (C) as the lowest possible grade for fats and oils could be considered in line with the FBDGs asking for moderate consumption of such products.

Limitations of the current study linked to the nature of the HelTH dataset have already been mentioned in the relevant sections. Although HelTh is the only available branded food composition database for Greece and covers an important part of the market, it is still in need of targeted expansions as for the case of oils and potentially novel foods like plant-based meat alternatives etc. Despite, its gaps the use of branded food composition databases is linked with substantial improvement in the relevance of the results for the consumer and the food industry as it is a direct reflection of the marketplace as compared to analyses performed on generic food composition data (31, 32).

This study also faced issues with missing data, especially for positive nutrients like protein and fiber. Although data completeness is relatively high for protein, fiber is only declared in food categories that are relevant or in foods that carry a nutrition claim for the specific nutrient (19). In the case of missing nutrient data, those were common among traditional artisanal foods that are not required by the regulation to carry a full nutritional declaration or due to the inability of the researchers to obtain access to the physical packaging of the foods. As described earlier, HelTH obtained data from products sold on e-shops of large supermarket chains. Often foods were missing clear images or images altogether from the nutritional declaration, in some cases those data have been added to the database through sampling in the physical supermarket but this process is still ongoing. The choice to impute positive nutrients with zero was merely of a mathematical nature. Imputation with zero for the positive nutrients was only likely to underestimate a food’s performance and that was decided to be the safest and prudent approach. However, the wider implementation of Nutri-Score as a FOP scheme is likely to resolve the data completeness issue as more manufactures would be displaying positive nutrients included in the Nutri-Score algorithm.

The hardest part of the Nutri-Score calculation is the calculation of the Fruits, Vegetables, Nuts, Pulses, and Oils component. This calculation has to be done manually and it is always linked to underestimation. Especially, in the context of the Mediterranean foodscape the importance of this component is vital as both national and Mediterranean Diet guidelines suggest that foods that contain vegetables or pulses and use olive oil as their main fat should be preferred (21, 33). Another area of importance is the use of dietary fiber as a proxy for wholegrain cereal content. As fiber and wholegrain content do not always correlate, the addition of wholegrain content as part of the Nutri-Score algorithm could be considered (34).

In the case of testing the alignment with existing FBDGs there are additional limitations to be considered. These include issues like the misalignment of the food categories as mentioned in the guidelines as opposed to the food categories proposed by systems like Langual or FoodEx2. For example, although the guidelines considered fine bakery ware to be considered sweets from a food technology point of view these foods are more likely to be classified as cereal-based foods. Similarly, the guidelines often refer to decreased intake of trans-fatty acids and increased intake of fiber and wholegrains, however this information is not mandatory as part of the nutritional declaration in EU and is often missing or it needs to be manually estimated from the ingredients list.

Although, this work offers evidence on the alignment of Nutri-Score with national FBDGs, it is important for Nutri-Score to be tested against dietary patterns with a documented beneficial effect on health. The Mediterranean Diet Pyramid is such a pattern and its principles expand beyond the nutritional composition of foods to cover elements of locality, tradition, seasonality, culinary, and cultural elements. Testing the alignment of Nutri-Score with dietary patterns like the Mediterranean Diet would require targeted analysis and testing. Future work should aim to directly test the alignment of Nutri-Score with these guidelines as it will allow to answer questions around the type of reformulation that Nutri-Score will promote in the Mediterranean and whether the traditional cooking techniques will be favored and what the impact of Nutri-Score would be on the Mediterranean agri-food value chain.

## Conclusion

Overall, this study is the first to report the performance and discriminatory capacity of Nutri-Score in the Greek foodscape using a branded food composition database. It highlights an overall good discriminatory capacity and satisfactory agreement with the national FBDGs. However, the evaluation of an upcoming food policy requires further data on the consumer perception and likelihood for adoption, as well as an analysis of the alignment with the existing agricultural policies and agroeconomic strategies. After this complete description of the risks and benefits a roadmap of implementation could be developed.

## Data availability statement

The raw data supporting the conclusions of this article will be made available by the authors, without undue reservation.

## Author contributions

AV: formal analysis and writing—original draft preparation. AV and AK: investigation and data curation. MK: resources and supervision. All authors have conceptualization, methodology, writing—review and editing, read, and agreed to the published version of the manuscript.
